# The Impact of Proinflammatory M1 Macrophages on the Proliferation and Expression of Cyclin E2, Mitogen-Activated Protein Kinases 4 and 7 in Hepatocytes Isolated from a Diethylnitrosamine-Induced Hepatocellular Carcinoma Rat Model

**DOI:** 10.3390/molecules30173657

**Published:** 2025-09-08

**Authors:** Marta Wójcik, Luisa Pozzo, Andrea Vornoli, Vincenzo Longo, Anna Śmiech, Joanna Czerwik-Marcinkowska, Iwona Rozempolska-Rucińska, Agnieszka Chrapko, Szymon Zmorzynski

**Affiliations:** 1 Oncology Lab, Department of Epizootiology and Clinic of Infectious Diseases, Faculty of Veterinary Medicine, University of Life Sciences in Lublin, 20-612 Lublin, Poland; 2 Institute of Agricultural Biology and Biotechnology, National Research Council, 56124 Pisa, Italy; 3 Department of Pathomorphology and Forensic Medicine, Faculty of Veterinary Medicine, University of Life Sciences in Lublin, 20-612 Lublin, Poland; 4 Institute of Biology, Jan Kochanowski University, 25-369 Kielce, Poland; 5 Institute of Biological Basis of Animal Production, University of Life Sciences in Lublin, 20-950 Lublin, Poland; iwona.rucinska@up.lublin.pl; 6 Institute of Health Sciences, the J.P II Catholic University of Lublin, 20-708 Lublin, Poland; 7 Institute of Human Sciences, Faculty of Health Sciences, Academy of Zamosc, 22-400 Zamosc, Poland

**Keywords:** liver cancer, cyclins, kinases, hepatocellular carcinoma, animal model, rats, M1 macrophages, hepatocytes

## Abstract

Background: Hepatocellular carcinoma (HCC) is highly resistant to conventional therapies, highlighting the need for novel immunotherapeutic approaches. In the tumor microenvironment (TME), the role of proinflammatory M1 macrophages remains ambiguous. The proteins *Mapk4/7* and cyclin E2 (CE2, Ccne2) are crucial for regulating hepatocyte proliferation and may be important factors driving the development of HCC. This study aimed to investigate the effects of M1 macrophages on *CE2* and *Mapk4/7* expression, as well as hepatocyte proliferation, in a rat model of partial hepatectomy (PH) with or without diethylnitrosamine (DEN)-induced HCC. (2) Methods: Twenty female Wistar rats were assigned to nonneoplastic (PH) or neoplastic (PH/DEN) groups. Gene expression (*CE2*, *Mapk4/7*) was quantified via real-time PCR. (3) Results: Overexpression of *CE2* and increased proliferation were observed in PH/DEN hepatocytes, whereas exposure to proinflammatory M1 macrophages significantly reduced their proliferative activity. *Mapk4/7* expression patterns were modulated by the TME and significantly differ depending on macrophage activation status in both PH and PH/DEN-derived hepatocytes. (4) Conclusions: Our findings indicate that *CE2* expression is upregulated in PH/DEN cells, with a notable decrease in the presence of M1 macrophages. In contrast, compared with control macrophages, M1 macrophages did not significantly affect *Mapk4/7* expression.

## 1. Introduction

Hepatocellular carcinoma (HCC) is the most common type of primary liver cancer and is associated with high morbidity and mortality rates [1,2,3]. Despite significant advances in the management of HCC, the prognosis after local therapies and surgical resection remains poor [4].

The tumor microenvironment (TME) consists of malignant cells and a variety of normal cell types [5]. Tumor-transformed hepatocytes exhibit increased anaerobic glycolysis and secrete signaling molecules, including L-6, tumor necrosis factor alpha (TNF-α), and exosomal miRNAs, into the microenvironment [6,7,8,9]. Various factors can influence the TME through distinct mechanisms, notably by modulating macrophage activity. Within the TME, immune cells with diverse phenotypes are present, including both proinflammatory M1 and anti-inflammatory M2 macrophages [10]. Tumors tend to promote the M2 phenotype of macrophages (i.e., immunosuppressive, promoting angiogenesis and tumor growth), precisely to avoid the immune response [11]. Nevertheless, some inflammatory signals released from necrotic tumor areas can temporarily activate macrophages toward the M1 phenotype (proinflammatory), especially in the very early stages of the antitumor response [12]. Although M2 macrophages (Mf-M2) are well recognized for their protumor role, the function of M1 macrophages (Mf-M1) in HCC development is complex and context dependent [13]. On the one hand, M1 macrophages are thought to be tumoricidal, through the release of inflammatory cytokines, reactive nitrogen intermediates (RNI), and reactive oxygen species (ROS) [14]. From another perspective, these macrophages promote tumorigenesis and tumor progression [13].

It has been suggested that cell proliferation is directly associated with carcinogenesis [15]. During HCC development, an increase in the rate of proliferation is one of the earliest phenotypic changes detected in preneoplastic and neoplastic hepatocytes [16]. Thus, cell proliferation is a useful parameter for predicting the aggressiveness of HCC cells. Mapk4 and Mapk7 (Erk5, Bmk1) belong to the mitogen-activated protein kinase (Mapk) subfamily, which regulates many cellular processes, including proliferation, differentiation, and the stress response [17,18]. They are critical in the passage of cells through cell cycle checkpoints. Mapk4/7 kinases can modulate cyclin expression. Cyclin E2 (CE2, Ccne2) binds and activates its kinase partner, the cyclin-dependent kinase 2 (Cdk2) [19]. Thus, the formed complexes phosphorylate proteins that govern cell cycle progression, as well as proteins involved in histone biosynthesis, centrosome duplication, and the firing of DNA replication origins [20]. Through the phosphorylation of regulatory proteins, Mapk4/7 affects the activity of CE2/Cdk2 complexes, facilitating cell cycle progression [21]. In HCC, increased Mapk activity is often associated with increased tumor cell proliferation [18,22]. These findings suggest that increased activity of Mapks, including Mapk4 and Mapk7, may contribute to increased proliferation of cancer cells in HCC. *CE2* overexpression promotes uncontrolled cell division and tumor progression [23]. During the cell cycle, the relevant mechanism that drives cells from the G_0_/G_1_ phase into the S phase is controlled by CE2 [19]. For HCC, many authors have indicated a strong relationship between the expression of *CE2* and the proliferative activity of neoplastic liver cells [23,24,25]. Cyclin E overexpression is observed in most HCC cases and has been shown to be correlated with poor clinical outcomes [25]. Collectively, these findings suggest that CE2 intensifies cell proliferation, driving tumorigenic processes during HCC development [26].

Considering the above information, we analyzed the effects of proinflammatory M1 macrophages on the expression of *CE2* and *Mapk4/7* in hepatocytes isolated from a rat model of diethylnitrosamine-induced (DEN-induced) hepatocellular carcinoma. DEN is a very well-known chemical carcinogen that is widely used in experimental models to induce HCC in rodents [27]. It mimics the processes that occur in humans with chronic liver damage (e.g., alcohol, hepatitis B/C), but in an accelerated and controlled manner [28]. To investigate the correlation between *CE2*, *Mapk4/7* and neoplastic hepatocyte apoptosis, we employed a well-known and highly reproducible rat model of DEN-induced hepatocarcinogenesis. Moreover, considering the tumorigenic activity of Mf-M1, we sought to analyze their influence on *CE2* expression, as well as on *Mapk4/7* and the proliferative activity of neoplastic rat hepatocytes. To achieve this goal, we used the dynamic flow Quasi-Vivo System, which allows the coculture of different cell types, thereby allowing the analysis of their interactions with each other. To the authors’ knowledge, the proposed research has not previously been conducted to this extent.

## 2. Results

The present study concerns the analysis of *CE2* and *Mapk4/7* expression in rat hepatocytes after partial hepatectomy (PH) and PH/DEN. In addition, we analyzed (I) liver function via the following serum markers: alanine aminotransferase (ALT), aspartate aminotransferase (AST), γ-glutamyltransferase (GGT) and alkaline phosphatase (AP); (II) morphological and histological changes in the liver; (III) alpha-fetoprotein (AFP) levels in hepatocytes (as a marker of HCC); and (IV) changes in the morphology of hepatocytes cultured with Mf and Mf-M1.

### 2.1. Body Weight and Blood Biochemical Analyses

Compared with animals subjected to partial hepatectomy alone (PH), those subjected to partial hepatectomy combined with diethylnitrosamine administration (PH/DEN) presented a significantly reduced growth rate relative. At the time of sacrifice, the body weights of the PH/DEN-treated rats were approximately 18% lower than those of the PH-treated rats, and this difference was statistically significant. Additionally, the plasma levels of AST, ALT, ALP, and GGT were significantly elevated in the PH/DEN group compared with those in the PH-only group (Table 1).

### 2.2. Histological Examination of PH/DEN-Induced Livers

**Figure 1:**
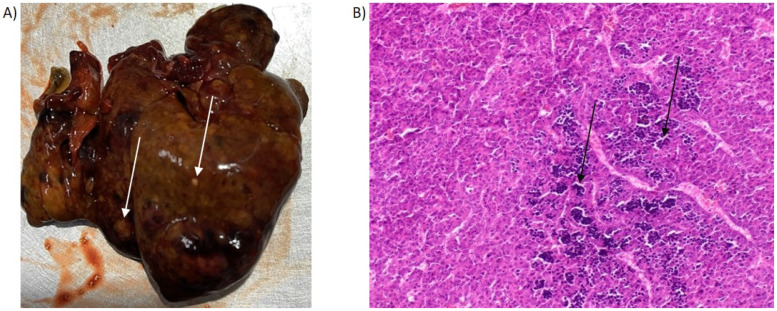
Representative macroscopic and histological features of livers from PH/DEN-treated rats. (**A**) Multiple white nodules and hemorrhages were macroscopically observed. (**B**) Histological sections revealed an irregular cellular architecture, nuclear pleomorphism with multinucleation, inflammatory cell infiltration, and mild congestion of the hepatic sinusoids (total magnification ×200). The arrows indicate the affected regions.

Chronic liver damage and regions exhibiting cellular atypia were characteristic features observed in liver sections from PH/DEN-treated rats (Figure 1). The most frequently identified histopathological changes included lymphocytic infiltration, hepatocytes with enlarged nuclei, and atypical cellular morphology.

### 2.3. Alpha-Fetoprotein Levels in PH- and PH/DEN-Derived Hepatocytes

The identity of the cultured neoplastic hepatocytes was confirmed by measuring the concentration of AFP, a standard and widely accepted biomarker of HCC. Elevated AFP levels are commonly associated with HCC. In our study, the AFP concentrations in the PH/DEN-derived cell lysates were approximately sixfold greater than those in the PH group, reaching 61 ± 5.7 ng/mg protein versus 10 ± 2.1 ng/mg protein, respectively (*p* < 0.001).

### 2.4. Phenotypes of Primary PH- and PH/DEN-Derived Hepatocyte Cultures

**Figure 2:**
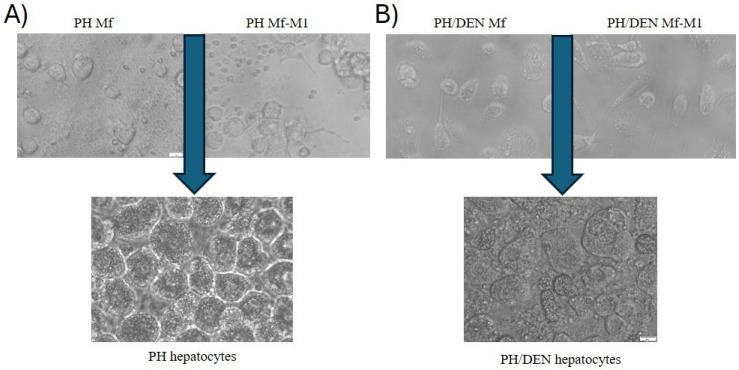
Morphological phase-contrast characteristics of the isolated and cultured macrophages and hepatocytes. (**A**) Nonstimulated (Mf) and M1 (Mf-M1) macrophages cocultured with PH hepatocytes. Mf: rounded, lightly adherent. Mf-M1: more flattened, more intensely stretched, sometimes with pseudopodia indicative of activation; (**B**) nonstimulated (Mf) and M1 (Mf-M1) macrophages cocultured with PH/DEN hepatocytes. The above morphological changes in Mf-M1 cells occur together with their proinflammatory polarization, as reflected by the increase in ROS, RNI, and proinflammatory cytokine (TNFα, IL-6) levels. A more significant response to BBG was detected with respect to PH/DEN-derived Mf-M1 (data not published).

PH-derived hepatocytes adhered to the culture dish within 24 h and, by 72 h, reached confluence, exhibiting the characteristic polygonal morphology of mature hepatocytes (Figure 2A). These cells presented well-defined intercellular boundaries. In contrast, PH/DEN-derived hepatocytes proliferated in culture without forming a regular monolayer (Figure 2B). These cells lacked the typical polygonal morphology of differentiated hepatocytes and displayed a heterogeneous phenotype, with populations of both smaller and larger cells. Furthermore, after 72 h, the PH/DEN hepatocytes began to aggregate and form small colonies.

### 2.5. CE2 Expression Under Nonneoplastic and Neoplastic Conditions

**Figure 3:**
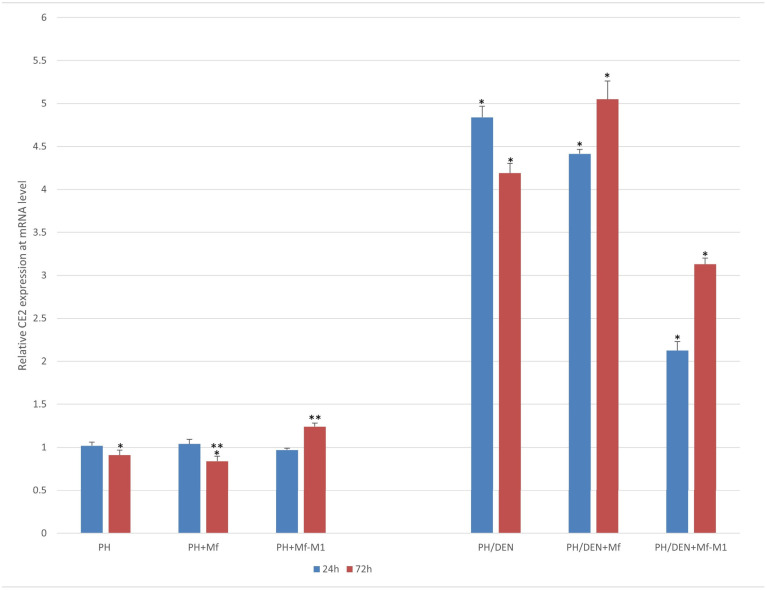
The expression of *CE2* taking into account time and the tumor microenvironment. PH—partial hepatectomy, Mf—macrophages, Mf-M1—M1 macrophages, DEN—diethylnitrosamine; *, ** statistically significant differences.

*CE2* expression at the mRNA level was detectable in hepatocytes derived from both the PH and PH/DEN models. In PH-derived hepatocytes, *CE2* expression remained unchanged after 24 h of culture with mRNA levels close to 1.0, irrespective of coculture with either nonstimulated macrophages (Mf) (*p* = 0.106) or proinflammatory macrophages (Mf-M1) (*p* = 0.186) (Figure 3). However, after 72 h, PH hepatocytes cocultured with Mf presented significantly lower *CE2* expression than PH hepatocytes alone (*p* < 0.0001) and PH+Mf-M1 hepatocytes (*p* < 0.0001) (Figure 3). *CE2* was overexpressed in PH/DEN cells after 24 h and 72 h. An increase in *CE2* was detected after 24 h and 72 h in PH/DEN-hepatocytes cultured alone or in combination with nonstimulated macrophages (Mf) (Figure 3)—PH/DEN vs. PH/DEN+Mf, *p* < 0.001 and *p* < 0.001, respectively. When these cells were exposed to proinflammatory Mf-M1, the expression of *CE2* decreased compared with that in the PH/DEN and PH/DEN+Mf groups after 24 h (*p* < 0.001 and *p* < 0.001) and 72 h (*p* = 0.002 and *p* = 0.01), respectively.

### 2.6. Mapk4 Expression Under Nonneoplastic and Neoplastic Conditions

**Figure 4:**
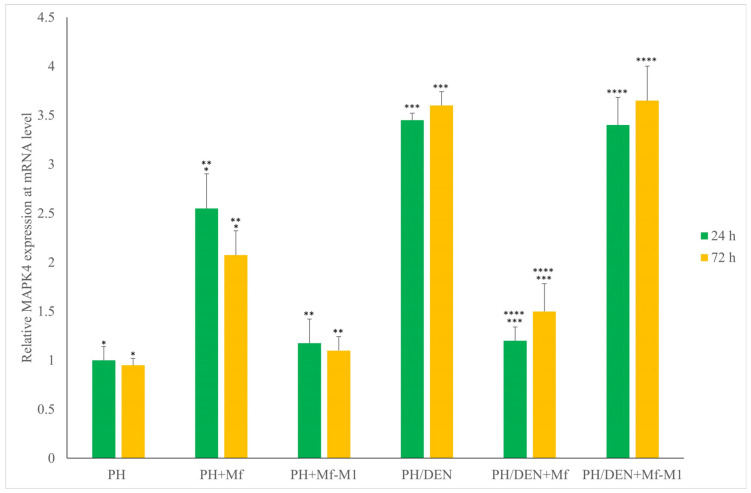
The expression of *Mapk4* taking into account time and the tumor microenvironment. PH—partial hepatectomy, Mf—macrophages, Mf-M1—M1 macrophages, DEN—diethylnitrosamine; *, **, ***, and **** indicate statistically significant differences.

Compared with those of PH and PH/DEN hepatocytes, the presence of M1 macrophages did not significantly alter *Mapk4* levels after 24 or 72 h (Figure 4). After 24 h, statistically significant differences in *Mapk4* expression were detected between PH and PH+Mf (*p* = 0.028), as well as between PH+Mf and PH+Mf-M1 (*p* = 0.045). Comparable statistically significant differences were also noted after 72 h. In the PH/DEN model, significant differences were detected at both 24 and 72 h between PH/DEN and PH/DEN+Mf (*p* = 0.002 and *p* = 0.01, respectively), as well as between PH/DEN+Mf and PH/DEN+Mf-M1 (*p* = 0.01 and *p* = 0.02, respectively).

### 2.7. Mapk7 Expression Under Nonneoplastic and Neoplastic Conditions

**Figure 5:**
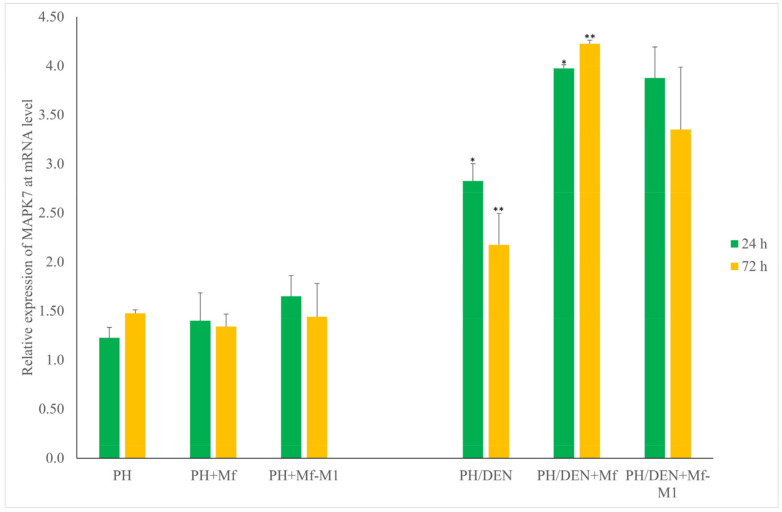
The expression of *Mapk7* taking into account time and the tumor microenvironment. PH—partial hepatectomy, Mf—macrophages, Mf-M1—M1 macrophages, DEN—diethylnitrosamine; *, ** statistically significant differences.

For *Mapk7* expression analysis, significant differences were noted only between the PH/DEN and PH/DEN+Mf groups at 24 h and 72 h (*p* = 0.01 and *p* = 0.01, respectively) (Figure 5). No significant differences were found in the other comparisons.

### 2.8. Hepatocyte Proliferation Analysis

**Figure 6:**
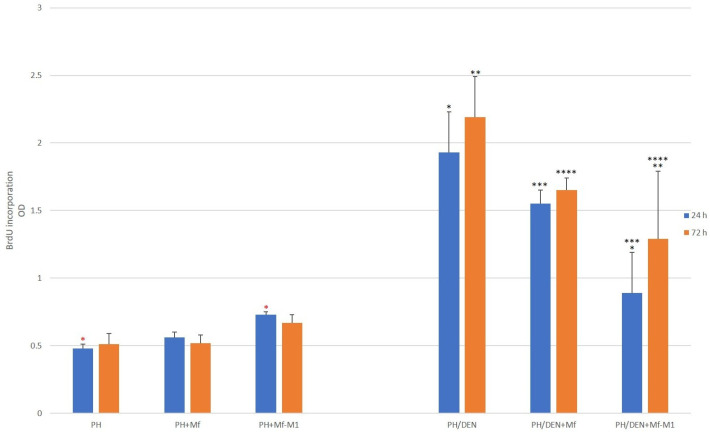
Proliferative activity of PH- and PH/DEN-hepatocytes assessed by BrdU incorporation; *, *, **, ***, **** statistically significant differences.

The proliferative activity of PH-obtained hepatocytes increased from 0.48 ± 0.03 (PH alone) to 0.73 ± 0.02 (PH-Mf+M1) after 24 h of culture, *p* < 0.001 (Figure 6). PH-hepatocyte proliferation, influenced by both Mf and Mf-M1, slightly increased with increasing culture time. However, these results were not statistically significant. In contrast, PH/DEN-hepatocytes showed proliferative activity throughout the entire incubation period, reaching the highest values under conditions without the influence of macrophages after 24 h and 72 h, with values of 1.93 ± 0.03 and 2.19 ± 0.3, respectively. Compared with that of nonstimulated Mf, the exposure of PH/DEN-hepatocytes to activated or nonstimulated Mf resulted in decreased proliferation. When PH/DEN cells were cocultured with proinflammatory Mf-M1 cells, the proliferation of neoplastic liver cells was significantly inhibited (*p* ≤ 0.05), with values of 0.89 ± 0.3 after 24 h (*p* = 0.001) and 1.29 ± 0.5 after 72 h (*p* = 0.01).

### 2.9. Apoptosis Analysis

After 24 h, the degree of apoptosis in PH-derived hepatocytes was similar between the PH and PH+Mf groups, whereas compared with the PH controls, the PH+Mf-M1 group presented a significant increase in the number of apoptotic cells (Table 2). The presence of M1-polarized macrophages markedly increased the number of apoptotic cells at 24 h in both the PH and PH/DEN models. A similar trend was observed at 72 h; however, the difference between PH and PH+Mf-M1 was not statistically significant. Nonstimulated macrophages (Mf) did not significantly affect apoptosis in PH-hepatocyte cultures at either 24 or 72 h (Table 2). After 24 h, a significant difference (*p* < 0.001) was observed between the PH+Mf and PH+Mf-M1 groups. However, after 72 h, this difference was not statistically significant (*p* = 0.067). Significant differences were observed between the PH/DEN+Mf and PH/DEN+Mf-M1 groups at both 24 and 72 h (*p* < 0.001).

### 2.10. Correlation Analysis

When PH/DEN-hepatocytes were influenced by nonstimulated Mf, a strong positive correlation between the proliferative activity of PH/DEN-hepatocytes and *CE2* expression was observed (r = 0.92, *p* < 0.001 after 72 h). The exposure of neoplastic hepatocytes to proinflammatory Mf-M1 macrophages was associated with a weakening of this correlation (r = 0.61, *p* = 0.06 after 72 h). After 24 h, a significant strong negative correlation was observed between apoptosis and *CE2* expression (Table 3). Proliferative activity was significantly correlated with apoptosis (*p* = 0.02) and *CE2* expression (*p* < 0.001). Other correlations, including those between apoptosis and *Mapk4* or *Mapk7*, were not statistically significant (*p* > 0.05), although *Mapk7* showed a positive trend. After 72 h, significant correlations were observed between proliferative activity and apoptosis (*p* < 0.001), *CE2* expression (*p* < 0.001), and *Mapk4* expression (*p* = 0.04). In the case of the other analyzed parameters, no statistically significant correlations were observed.

## 3. Discussion

The influence of different factors on liver regeneration and tumorigenesis in rats was investigated via two distinct models: a nonneoplastic model involving partial hepatectomy (PH), and a neoplastic model combining partial hepatectomy with diethylnitrosamine treatment (PH/DEN). Hepatocytes and monocytes derived from rat blood were isolated and cultured, after which they were subjected to macrophage polarization with barley β-glucan (BBG). The cells were cultured under static conditions and in a quasi-vivo perfusion system allowing coculture of hepatocytes and macrophages. We also analyzed *CE2* and *Mapk4/7* gene expression at the mRNA level, cell proliferation, AFP levels, and biochemical indices of liver function. In addition, histopathological evaluation of liver tissue was performed. The goal was to understand the mechanisms of regenerative and tumorigenic responses in the liver and the influence of the cellular microenvironment on these processes.

The PH/DEN model is commonly used to study hepatocarcinogenesis: it combines chemical DNA damage with the process of liver regeneration after partial hepatectomy, which faithfully reflects the mechanisms of liver cancer development in humans [29]. In our study, the PH/DEN model influenced both body weight and liver function markers. Consistent with our findings, Kirsch et al. and Maciejewska et al. reported reduced weight gain along with elevated liver enzyme activities [30,31]. Chronic lesions and areas showing features of cellular atypia in the PH/DEN model from the analyzed liver specimens were observed. The presence of these features indicates active inflammatory processes and dysplastic changes in hepatic cells, which are consistent with observations in the literature concerning the early stages of HCC [29,32,33,34].

The uncontrolled cell division driven by dysregulated cyclins and kinases, alongside the high rates of recurrence and metastasis, renders HCC one of the most aggressive cancers globally. In our study, proinflammatory M1-type macrophages polarized with BBG were shown to affect hepatocyte proliferation in a DEN-induced liver cancer model. Unregulated cell proliferation plays a central role in the development and progression of hepatocellular carcinoma and serves as a key indicator for assessing the biological aggressiveness of HCC [15]. Our data demonstrated high proliferative activity of neoplastic hepatocytes isolated from a PH/DEN-induced HCC rat model, which is consistent with findings reported in the literature [35,36]. Macrophages are tissue-resident phagocytes and antigen-presenting cells that differentiate from circulating peripheral blood monocytes. Stimulated macrophages of different phenotypes are routinely classified into M1 macrophages and M2 macrophages [37]. Stimulated M1 macrophages are immune effector cells with an acute inflammatory phenotype. M1 activation is triggered by interferon gamma, lipopolysaccharide, TNF-α and ß-glucan and is mediated by several signal transduction pathways involving a signal transducer and activator of transcription (STAT), as well as nuclear factor kappa-light-chain enhancer of activated B cells (NFkB) [38,39]. These transcription factors stimulate the production of microbicidal agents, including ROS and nitric oxide, while simultaneously promoting downstream inflammatory immune responses by increasing antigen presentation capacity and driving the activation of T helper 1 (Th1) cells via cytokines such as IL-12. In addition to their proinflammatory activity, M1 macrophages constitute one of the major populations of tumor-infiltrating immune cells and have well-documented functions in the regulation of the antitumor immune response [40,41]. In line with these findings, our results indicate that the proliferative activity of PH/DEN-derived hepatocytes was effectively inhibited despite exposure to Mf-M1. Conversely, when PH/DEN-hepatocytes were influenced by nonstimulated macrophages (Mf), increased cell proliferation was observed. Our findings are consistent with those reported by numerous researchers, including Zhou et al., 2020 [42] who reported that Mf-M1 exerts antitumor effects by suppressing proliferation and promoting apoptosis in HCC cells [42]. The predominance of M2 macrophages over M1 macrophages within the TME is associated with poor prognosis in patients with HCC [43,44]. Conversely, increasing evidence suggests that M1 macrophages play dual roles in hepatocarcinogenesis, exhibiting not only antitumor but also protumor functions. M1 macrophages can play a prometastatic role since they induce epithelial–mesenchymal transition (EMT) in pancreatic cancer cells and enhance the motility of HCC cells [45,46]. Unfortunately, it is still unknown whether M1 macrophages promote or inhibit hepatocarcinogenesis. A deep understanding of the role of M1 macrophages in HCC is vital for the development of novel targeted immunotherapies.

Tumor cells under conditions of severe inflammation (e.g., those generated by M1 macrophages) can upregulate the expression of cyclins (including *CE2*) as an escape mechanism from cytotoxic stress through the production of ROS and reactive nitrogen species [47,48,49,50]. M1 macrophages may paradoxically increase cyclin expression in cancer cells as an adaptive response [51]. M1-induced inflammatory stress can lead to tumor reprogramming, including a transient increase in cyclin expression [47,49]. Our results demonstrate that PH/DEN hepatocytes exhibit increased mRNA expression of *CE2*, which coincides with enhanced proliferative activity. Therefore, our findings support these observations, highlighting the role of *CE2* in cell cycle progression. Several lines of evidence have revealed that cyclins, including *CE2*, drive cell proliferation by initiating DNA replication and activating cell division kinases [52,53]. Many authors also point to a relationship between *CE2* overexpression and increased cell proliferation during HCC development [26,54]. A similar relationship was also observed in our research.

In our study changes in the expression of cell cycle-related genes such as *CE2*, *Mapk4* and *Mapk7* kinases were observed. Proteins encoded by these genes are important for regulating cancer cell proliferation. Mapk4 and Mapk7 can affect cell proliferation through different pathways and mechanisms of action. There is no direct activation cascade between these kinases—Mapk4 does not activate Mapk7 and vice versa—although they can function in parallel in the cell, regulating different processes [18,55]. Mapk4 is a kinase that can activate the AKT/mTOR signaling cascade, thereby promoting cell cycle progression and proliferation. This cascade plays a key role in regulating the synthesis of cyclins, including *CE2*, which governs the transition from the G1 phase to the S phase [56]. Mapk4 indirectly modulates the inflammatory functions of macrophages. The literature suggests that Mapk4 may function to suppress excessive proinflammatory signaling [18]. Our results revealed that increased *Mapk4* expression in the TME in the presence of M1 macrophages was indicative of a dynamic cellular response to inflammatory stress. Our findings suggest that M1 macrophages, despite their proinflammatory profile, may paradoxically facilitate cancer cell adaptation to stress and increase their survival. After PH, hepatocytes undergo regeneration and are receptive to pro-regenerative cytokines (e.g., IL-6, TNF-α, and IL-1β) secreted by macrophages [57]. These signals can activate the JAK/STAT and NF-κB pathways, which in turn increase the transcription of genes such as *Mapk4* that support proliferation and stress adaptation [57]. In contrast, in the PH/DEN group, hepatocytes were exposed to carcinogen-induced genomic stress and chronic inflammation, which reprogrammed macrophages toward a tumor-promoting phenotype [58]. In cells within the tumor microenvironment, Mapk4 can be upregulated as a protective mechanism against the cytotoxic effects of M1 macrophages, for example, by activating prosurvival signaling pathways. Another kinase we studied was Mapk7, also known as Erk5. It is a classical kinase that is activated by various stress and growth factors. Mapk7 plays an important role in the processes of cell proliferation, differentiation and stress response. Its activation occurs as part of the classical signaling cascade. A distinctive feature of Mapk7 is the presence of a unique transcriptional domain, which allows it to also perform functions in the cell nucleus, affecting gene expression [55].

The presence of macrophages in the PH/DEN model may cause an increase in *Mapk7* expression due to the role of this kinase in the cellular response to inflammatory factors and environmental stress. Macrophages, especially those with a proinflammatory phenotype (e.g., M1), secrete cytokines and inflammatory mediators that can activate signaling pathways in cancer cells and the TME, including Mapk7 [59]. Its activation in such an environment promotes the adaptation of cancer cells to stress, supports their survival and may promote tumor progression [60]. M1 macrophages, which are characterized by a proinflammatory phenotype, can stimulate the expression of this kinase in target cells. M1 macrophages are activated in response to stress and inflammatory signals, while M1 macrophages produce various proinflammatory cytokines—such as TNF-α, IL-1β, and IL-6—that trigger signaling pathways, resulting in increased expression and activity of these cytokines [59,61]. In our study, no statistically significant increase in *Mapk7* expression was detected in the PH/DEN Mf-M1 model. Similarly, a study on a DEN-induced HCC model reported that Kupffer cells (KCs) and monocyte-derived macrophages (Mo-Mφs) presented distinct gene expression profiles, yet no significant alterations in *Mapk7* expression were observed in response to tumor-associated inflammation [62]. Notably, these studies did not observe differences in the expression of Mf-M1 markers, such as CD68, compared with Mf-M2 markers, suggesting that macrophage activation in this model does not lead to a marked change in *Mapk7* expression [62]. Fan and colleagues reported comparable findings in a rat model of monocrotaline-induced pulmonary arterial hypertension [63]. Therefore, the lack of a statistically significant increase in *Mapk7* expression in our study may be consistent with the results of other studies suggesting that the activation of M1 macrophages in the PH/DEN model does not lead to a marked change in the expression of this kinase.

This study has several limitations. First, the expression of *CE2* and *Mapk4/7* was assessed solely at the mRNA level, without complementary protein-level analysis. Second, the neoplastic hepatocyte phenotype was not validated via flow cytometry. Third, evaluation of the hepatocyte cell cycle via flow cytometry was not performed, which would have enabled correlation analyses between kinase and cytokine expression and cell cycle phases. Fourth, ultrastructural analysis of the hepatocyte phenotype via electron microscopy was not conducted, limiting the assessment of intracellular changes, such as alterations in the nucleus, mitochondria, or cytosol. Fifth, the study relied solely on gene expression data, without complementary functional assays (such as cell cycle analysis, apoptosis markers, macrophage phenotypes, or phagocytic capacity), limiting mechanistic interpretation. Moreover, monocytes isolated from PH and PH/DEN rats may differ significantly, as DEN exposure can alter monocyte activation and differentiation. However, the specific impact of DEN on monocytes and their derived macrophage subsets (M1/M2) has not been addressed, limiting interpretation of the immunological findings. Finally, the relatively small sample size reduces the statistical power of the findings. Despite these limitations, this study represents the first comprehensive analysis of *CE2* and *Mapk4/7* expression in an HCC model incorporating Mf and Mf-M1 cells.

In summary, we investigated the inhibitory effect of Mf-M1 on the proliferation of neoplastic hepatocytes isolated from experimentally induced HCC in rats. However, we also concluded that the short-term exposure of PH/DEN-hepatocytes to Mf-M1 seems to be insufficient to effectively inhibit the proliferation of these neoplastic cells. Thus, the influence of M1 macrophages during HCC development should be further explored.

## 4. Materials and Methods

The study used animal experiments (including nonneoplastic and neoplastic groups) and then cell cultures (of hepatocytes and blood-derived monocytes) were conducted. The research was approved by the Ethics Committee for Animal Experimentation at the University of Life Sciences (Lublin, Poland; approval number 81/2015).

### 4.1. Reagents

BBG, collagenase, DEN, dexamethasone, Dulbecco’s modified Eagle’s medium (DMEM), fetal bovine serum, Ham’s F-12 medium, insulin, Krebs-Ringer buffer, nystatin, phosphate-buffered saline (PBS), penicillin/streptomycin, trypan blue, TRI reagent and nuclease-free water were all supplied by Sigma-Aldrich (Darmstadt, Germany). Other chemicals and materials: RevertAid First Strand cDNA Synthesis Kit, AmpliTaq Gold DNA polymerase, forward and reverse primers and FAM/MGB probes for real-time PCR, 96-well plates with optical covers (Thermo Fischer Scientific, Waltham, MA, USA), Lymphoprep (Nyegaard & Co, AS, Oslo, Norway) and 5-bromo-2′-deoxyuridine (BrdU) (Abcam, Eugene, OR, USA) were used.

### 4.2. Animal Model

**Figure 7:**
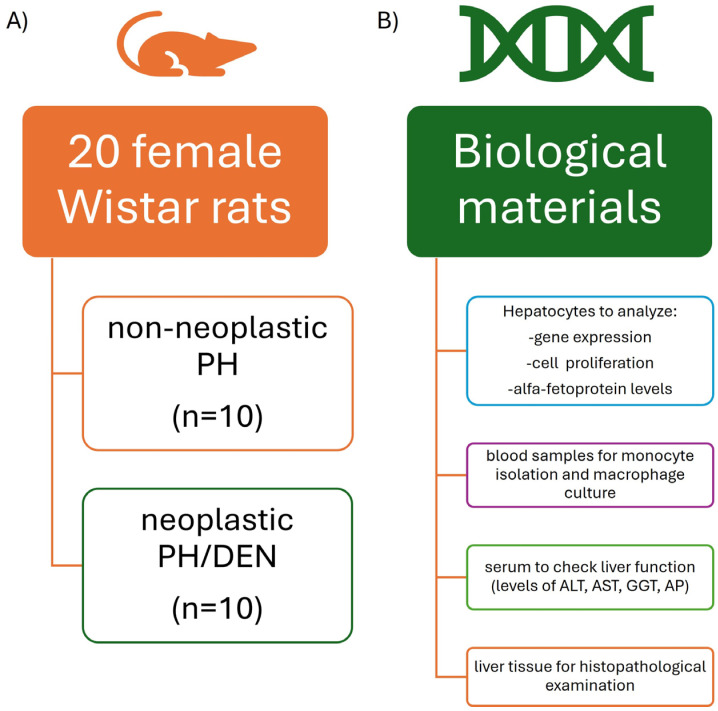
Animal groups (**A**) and samples(**B**) used in the experiment.

Experimental design: A total of 20 female Wistar rats (weight, 200–220 g) were used in the present study (Figure 7). The rats were obtained from the Centre of Experimental Medicine, The Medical University of Bialystok (Bialystok, Poland). The animals were housed with a 12 h light/dark cycle and had free access to drinking water and standard rat pellet food [64]. After a 1-week acclimatization period, 10-week-old rats were randomly assigned to two groups (10 animals per group): (I) the nonneoplastic PH group, which underwent partial hepatectomy (PH) and received PBS (0.005%) in the drinking water (*n* = 10); and (II) the neoplastic PH/DEN group, in which a two-step hepatocarcinogenesis model was induced according to the Solt–Farber protocol (*n* = 10) [65]. Rapid emergence of carcinogen-induced hyperplastic lesions in a new model for the sequential analysis of liver carcinogenesis. Following a 7-day acclimatization period, two-thirds partial hepatectomy (PH) was performed on all the rats in each group via the Higgins and Anderson method, which involved excision of the left lateral (~38%) and right (~30%) lobes, as previously described [66]. Seven days after PH, DEN was added to the drinking water of the neoplastic group of rats which were supplied ad libitum for 10 weeks. An exploratory laparotomy was then performed, and the development of tumors on the liver surface was confirmed. The nonneoplastic group was administered PBS in the drinking water.

### 4.3. Hepatocyte Isolation and Treatment

After 10 weeks, the rats were anesthetized with a mixture of ketamine and xylazine (90 mg/kg and 10 mg/kg, respectively), subjected to midline laparotomy to cannulate the portal vein, and perfused with Krebs-Ringer buffer, as described previously [67,68]. After filtration and centrifugation, the cell viability was determined via trypan blue exclusion and ranged from 75 to 80%. The cells were dispersed in DMEM/Ham’s F-12 medium (1:1 *v*/*v*) containing 39 ng/mL dexamethasone, 0.5 U/mL insulin, 100 U/mL penicillin, 0.1 mg/mL streptomycin, 100 U/mL nystatin and 10% fetal bovine serum. To prepare for Quasi-Vivo system analysis, hepatocytes were cultured at a density of 1 × 10^6^ cells/mL on glass membranes in 24-well plates and incubated under standard conditions (37 °C, 5% CO_2_) for 48 h to allow complete adhesion.

### 4.4. Isolation of Rat Blood-Derived Monocytes

Blood was taken from each animal during the experimental procedure but before liver perfusion (Figure 7). The mononuclear cells were isolated via Lymphoprep density-gradient centrifugation [14]. After being counted by an R1 Automated Cell Counter (Olympus, Warsaw, Poland) (cell viability >80%), the cells were plated into wells of a 24-well plate at a density of 2.5 × 10^5^ cells/mL and cultured at 37 °C with 5% CO_2_ for 24 h in DMEM/Ham’s F-12 (1:1 *v*/*v*) medium supplemented with 10% fetal bovine serum. The adherent cells were cultured for an additional 48 h to allow monocytes to mature into functional macrophages. After differentiation, the cells were polarized to the Mf-M1 phenotype with BBG at a concentration of 10 μg/mL. In a previous study, we used different concentrations of BBG (5–20 µg/mL) for polarization of rat monocytes, and we chose 10 μg/mL, which generated the best response of Mf to BBG after 24 h of exposure [14]. Every subsequent day of culture, the morphology of the macrophages was subjected to microscopic analysis as a qualitative assay. All functional assays of nonstimulated (Mf) and polarized (Mf-M1) macrophages were conducted as described previously [14].

### 4.5. Microenvironment Culture of Hepatocytes and Blood-Derived Monocytes

A commercially available perfusion chamber bioreactor (Quasi-Vivo, QV500, Kirkstall, UK) was used to keep the cells under perfusion. This system allows multiple cell types to be cultured in interconnected chambers under flow while sharing the same culture medium. Adherent hepatocytes and macrophages, previously plated on special glass membranes, were inserted into QV500 simple modular culture chambers. The chambers were subsequently connected in series with a peristaltic pump (ISMATEC, VWR International, Poland LTD., Gdańsk, Poland). The medium was pumped through the chambers at a rate of 150 µL/min. The hepatocytes were removed for analysis after 24 h and 72 h of incubation.

### 4.6. Biochemical Analysis of Liver Function

Liver function in serum was analyzed via ABX Pentra 400 a biochemical analyser (HORIBA Ltd., Kyoto, Japan) (Figure 7). Serum levels of alanine aminotransferase (ALT), aspartate aminotransferase (AST), γ-glutamyl transferase (GGT) and alkaline phosphatase (AP) were quantified according to the manufacturer’s protocol.

### 4.7. Histopathological Examination

Liver tissue was examined as described previously and analyzed via light microscopy (Eclipse E-600; Nikon Corporation, Tokyo, Japan) at ×200 magnification. The hepatic injuries were assessed according to the World Health Organization Histological Classification of Tumors [69].

### 4.8. Isolation of Total RNA from PH and PH/DEN Cells Cultured In Vitro

Total RNA was isolated from the cells via the TRI Reagent procedure according to Chomczyński [70]. The RNA pellet was subsequently resuspended in 75% ethanol. The purity of the total RNA samples was determined as the 260/280 nm OD ratio. The quality of these nucleic acids was checked via 2% agarose gel electrophoresis. The RNA was stored at −80 °C. The 260/280 ratios ranged from 1.95 to 2.06. The final RNA concentration was analyzed via the spectrophotometric method (CE 2021 UV/VIS Spectrophotometer, Cecil Instruments Limited, Cambridge, UK). The RNA samples were stored at −80 °C until further analysis.

### 4.9. Reverse Transcription

The reverse transcription reaction (RT-PCR) mixture was prepared after RNA isolation. RT-PCR was performed using a T100 Thermal Cycler™ (Bio-Rad, Hercules, CA, USA). Reverse transcription was carried out via a RevertAid First Strand cDNA Synthesis Kit. The cDNA obtained was stored at −20 °C until real-time PCR analysis.

### 4.10. Analysis of CE2 and Mapk4/7 Expression at the mRNA Level Using Real-Time PCR

The cDNA samples were amplified using AmpliTaq Gold DNA polymerase and the Applied Biosystem 7500 Real Time PCR (Fast System, Waltham, MA, USA). The reaction was carried out in 96-well plates with optical covers. To assess changes in the mRNA levels of the target gene relative to the reference gene Gadph (glyceraldehyde-3-phosphate dehydrogenase) using the relative quantification method, 5 ng of cDNA was amplified with rat-specific forward and reverse primers (each at 500 nM) and a fluorescently labeled probe (FAM/MGB, 200 nM). The assay ID and sources of primers and probes are given in Table 4. Every sample was assayed in duplicate, with expression being calculated according to the 2^−ΔΔCt^ method—normalized expression [71]. The expression values are presented as the logarithm of R to base 2, where R was calculated as follows: R = 2^−ΔΔCt^, ΔΔCt = ΔCt of the control −ΔCt of the analyzed gene, and every ΔCt = Ct of the analyzed gene—Ct of the endogenous control. The value of the normalized expression (R) in the range of 0.8–1.2 indicated a normal level of gene expression, R < 0.8 indicated low expression, and R > 1.2 indicated high expression [72,73].

### 4.11. Cell Proliferation Assay

The proliferation rates of PH and PH/DEN cells were assessed via an immunoenzymatic BrdU ELISA kit, following the manufacturer’s instructions. The immune complexes were detected at 450 nm using an ELx800™ ELISA reader (BioTek Instruments, Inc., Winooski, VT, USA).

### 4.12. Apoptosis Detection

Apoptosis was assessed using an Annexin V/FITC assay kit (Bio-Rad, California, USA) following the manufacturer’s instructions, as described previously [69]. Briefly, the cells were washed, centrifuged, stained with Annexin V-FITC and propidium iodide, and analyzed via a Coulter Epics XL flow cytometer (Beckman Coulter, Inc., Brea, CA, USA).

### 4.13. Alpha-Fetoprotein (AFP) ELISA

The protein level of AFP in the hepatocyte lysates was determined via an assay kit (Cloud-Clone Corp., Houston, TX, USA) according to the manufacturer’s instructions. The optical density (OD) was determined using a microplate reader (ELx800™ ELISA reader, BioTek Instruments, Inc., Winooski, VT, USA) at 450 nm. The concentration of AFP was analyzed on the basis of a standard curve. According to Wolf and Peschke, normal liver tissue, dysplastic foci and hyperplastic nodules do not express AFP [74].

### 4.14. Statistical Analysis

All experiments were repeated a minimum of three times with different batches of cell samples. The data are presented as the means ± standard deviations. The data were analyzed via one-way analysis of variance (ANOVA) or Student’s *t*-test as appropriate. When ANOVA indicated significant differences within groups, comparisons were made via Tukey’s post hoc test. Correlations between variables were assessed using Pearson’s correlation coefficient. We assumed a 5% error of inference and a related level of significance of *p* < 0.05, indicating the existence of statistically significant differences. Statistical analyses were performed using Statistica version. 13.3 (StatSoft) software.

## 5. Conclusions

The following conclusions can be drawn from our research: (I) rats subjected to PH/DEN treatment exhibited more pronounced liver damage than those subjected to partial hepatectomy alone; (II) PH/DEN samples presented characteristics of liver injury and neoplastic alterations; (III) significantly elevated AFP levels in PH/DEN cells compared with those in PH cells confirmed neoplastic transformation; (IV) hepatocytes isolated from the PH/DEN group exhibited altered morphology characterized by loss of typical cellular structure, heterogeneity in cell size, inability to form a regular monolayer, and loss of contact inhibition; (V) PH/DEN hepatocytes presented markedly greater overexpression of *CE2* than PH cells, reflecting their enhanced proliferative capacity linked to neoplastic transformation; (VI) *Mapk4/7* expression was not significantly affected by M1 macrophages; and (VII) PH/DEN hepatocytes exhibited significantly greater proliferation than PH cells, with the greatest proliferation observed in macrophage-free conditions. The presence of proinflammatory M1 macrophages markedly reduced proliferation and appeared to modulate the role of *CE2*, which strongly correlated with cell growth in the absence of macrophages.

In summary, the PH/DEN model in rats showed neoplastic transformation of hepatocytes, as evidenced by liver damage, dysplasia, elevated AFP, and *CE2* overexpression and loss of growth control, with M1 macrophages able to partially limit the proliferation of these cells, suggesting their potential anticancer effects.

## Figures and Tables

**Table 1:** Effects of partial hepatectomy and PH/DEN-induced hepatocarcinogenesis on body weight, and the serum levels of AST, ALT, AP and GGT.

Feature	PH-Rats (mean, ±SD)	PH/DEN Rats (mean, ±SD)
Initial weight (g)	203 ± 2.18	202 ± 8.2
Final weight (g)	312 ± 1.93	258 ± 9.2 *
AST (U/L)	105 ± 3.88	210.25 ± 19.04 *
ALT (U/L)	47.15 ± 1.09	77.65 ± 5.15 *
AP (U/L)	72.85 ± 1.34	142.75 ± 2.1 *
GGT (U/L)	5.15 ± 0.65	61.65 ± 9.08 *

SD—standard deviation; * *p* ≤ 0.05 vs. PH rats; AST—aspartate aminotransferase; ALT—alanine aminotransferase; AP—alkaline phosphatase; GGT—γ-glutamyl transferase; PH—partial hepatectomy; DEN—diethylnitrosamine.

**Table 2:** Apoptosis of hepatocytes (% of cells) under various culture conditions.

Hepatocytes	24 h (%, ±SD)	*p* Value	72 h (%, ±SD)	*p* Value
PH	13.14 ± 1.2	-	16.86 ± 3.5	-
PH and Mf	12.7 ± 1.8	*p* = 0.89 *	15.44 ± 2.5	*p* = 0.31 *
PH and Mf-M1	19.2 ± 2.9	*p* < 0.001 **	17.9 ± 3.1	*p* = 0.49 **
PH/DEN	1.5 ± 0.2	-	2.9 ± 0.7	-
PH/DEN and Mf	3.08 ± 0.5	*p* < 0.001 ***	5.94 ± 0.8	*p* < 0.001 ***
PH/DEN and Mf-M1	10.8 ± 1.4	*p* < 0.001 ****	15.8 ± 2.1	*p* < 0.001 ****

* PH vs. PH+Mf; ** PH vs. PH+Mf-M1; *** PH/DEN vs. PH+Mf; **** PH/DEN vs. PH+Mf-M1; SD—standard deviation.

**Table 3:** Correlation matrix between apoptosis and gene expression (*CE2*, *Mapk4*, *Mapk7*) in hepatocyte cultures. The values represent Pearson correlation coefficients (r).

**After 24 h**					
	Apoptosis	*CE2*	*Mapk4*	*Mapk7*	*Proliferative activity*
Apoptosis					
*CE2*	−0.94 *				
*Mapk4*	−0.39	0.33			
*Mapk7*	−0.64	0.71	0.36		
*Proliferative activity*	−0.88 *	0.98 *	0.37	0.36	
**After 72 h**					
Apoptosis					
*CE2*	−0.71				
*Mapk4*	−0.31	0.32			
*Mapk7*	−0.41	0.80	0.18		
*Proliferative activity*	−0.91 *	0.92 *	0.68 *	0.61	

* statistically significant values.

**Table 4:** Assay IDs of the primer/probe sets for the genes analyzed via real-time PCR.

Gene Name	Assay ID of Primer/Probe Set	References/Source
*CE2* (rat) Primers/probe set (FAM-MGB)	Rn01442731_m1	Life Technologies Poland LTD (Warszawa, Poland)
*Mapk4* (rat) Primers/probe set (FAM-MGB)	Rn01461524_m1	Life Technologies Poland LTD (Warszawa, Poland)
*Mapk7* (rat) Primers/probe set (FAM-MGB)	Rn01403106_m1	Life Technologies Poland LTD (Warszawa, Poland)
*Gadph* (rat) Primers/probe set (FAM-MGB)	Rn01775763_g1	Life Technologies Poland LTD (Warszawa, Poland)

## Data Availability

The data presented in this study are available upon request from the corresponding author owing to ethical restrictions related to the use of animal experimental data.

## Abbreviations

The following abbreviations are used in this manuscript:

AFP	alpha-fetoprotein
ALT	alanine aminotransferase
AP	alkaline phosphatase
BBG	barley β-glucan
BrdU	5-Bromo-2′-deoxyuridine
CDK2	cyclin-dependent kinase 2
CE2	cyclin E2
DEN	diethylnitrosamine
DMEM	Dulbecco’s modified Eagle’s medium
HCC	hepatocellular carcinoma
MAPK	mitogen-activated protein kinase
Mf	macrophages (control macrophages)
Mf-M1	M1 macrophages (cells with M1 phenotype)
Mf-M2	M2 macrophages (cells with M2 phenotype)
NFkB	nuclear factor kappa-light-chain enhancer of activated B cells
PBS	phosphate-buffered saline
PH	partial hepatectomy
ROS	reactive oxygen species
STAT	signal transducer and activator of transcription
TME	tumor microenvironment
TNF	tumor necrosis alpha
